# SARS-CoV-2 spike protein: flexibility as a new target for fighting infection

**DOI:** 10.1038/s41392-020-00369-3

**Published:** 2020-10-30

**Authors:** Ciro Leonardo Pierri

**Affiliations:** 1 Laboratory of Biochemistry; Molecular and Structural Biology; Department of Biosciences, Biotechnologies, Biopharmaceutics, University of Bari, Via E. Orabona, 4, 70125 Bari, Italy; 2 BROWSer S.r.l. (https://browser-bioinf.com/) c/o Department of Biosciences, Biotechnologies, Biopharmaceutics, University “Aldo Moro” of Bari, Via E. Orabona, 4, 70126 Bari, Italy

**Keywords:** Structural biology, Target identification

The recent study of Turoňová et al., is the first to combine cryo-electron tomography, subtomogram averaging, and molecular dynamics simulations for analyzing the structure of the SARS-CoV-2 spike protein in situ,^1^ i.e. while the virus interacts with tissue cultured cells.

The spike protein of the severe acute respiratory syndrome coronavirus 2 (SARS-CoV-2) is crucial for the infection because it mediates the virus entry into human host-cells, through interactions between the SARS-CoV-2 spike receptor-binding domain (RBD) and the human ACE2 receptor. Since spike proteins represent the major target of neutralizing antibodies, as observed for SARS-CoV-1 and other coronaviruses,^2–4^ understanding their conformational change mechanisms is necessary for developing new neutralizing antibodies and vaccines also for SARS-CoV-2.

Cryo-electron microscopy (cryo-EM) allowed to solve the structure of the spike protein of SARS-CoV-2 confirming that its prefusion conformation has the same overall trimeric structure observed in previous deposited cryo-EM structures of spike proteins from SARS-CoV-1 and other coronaviruses.^2–4^

Turoňová et al. also observed that the viral spike protein in situ was more heavily glycosylated than expected and occurred mostly in the closed prefusion conformation, compared to what observed with the recombinant spike proteins.^1^ Subtomogram averaging analysis resulted in a cryo-EM map of the spike head at 7.9Å resolution suggesting that about half of the spike protein was present in the fully closed prefusion conformation. A considerable fraction of the remaining subtomograms had one RBD (residues C336-E516, i.e., 6vbs.pdb numbering) exposed (in upward conformation).^1^

**Fig. 1:**
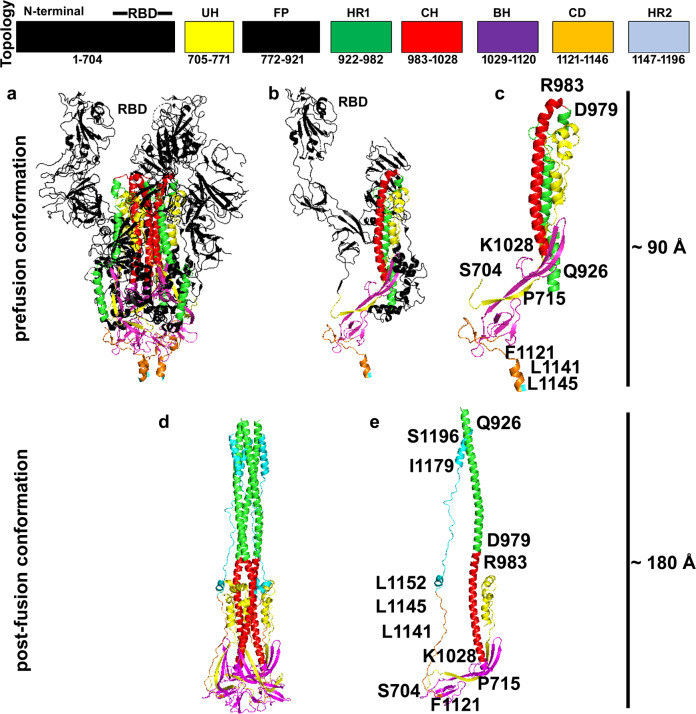
Protein regions of the SARS-CoV-2 spike protein involved in conformational changes determining the post-fusion conformation. Topology panel: SARS-CoV-2 spike protein block organization. Colors and residues numbering above the boxes reflect those reported in the 3D comparative model of SARS-CoV-2 spike protein^2^ reported below (YP_009724390.1 residues numbering). Abbreviations: RBD receptor-binding domain, located approximately at the end of the indicated N-terminal domain in the top portion of the prefusion conformation; UH upstream helix; FP the region hosting the fusion peptide; HR1 heptad repeat 1; CH central helix; BH β-hairpin region; CD connector domain; HR2 heptad repeat 2. **a**, **d** Lateral views of the SARS-CoV-2 spike protein trimer in pre-/post-fusion conformation, respectively, are reported in colored cartoon representation. Black cartoon protein regions in **a**, **b** indicate regions lost after cleavage events and/or not available in the available crystallized prefusion structures (according to ref. ^2^). **b**, **c**, **e** Lateral views of the SARS-CoV-2 spike monomer in pre-(**b**, **c**)/post(**e**)-fusion conformation, are reported in colored cartoons. Yellow (S704-I771), magenta (M1029-T1120) and red (R983-K1028) cartoon indicate the protein regions involved in few conformational changes, whereas green (Q926-S982) and orange (F1121-D1146) cartoon indicate protein regions involved in large conformational changes. Residues to be used as a reference for identifying quickly the cited protein regions are labeled. Cyan cartoon protein regions in **d**, **e** indicate the 1146–1197 protein region obtained by comparative modeling^2^ based on the crystallized structure of a similar coronavirus spike protein.^5^ Leucine residues reported in (**c**, **e**) are those of the first hinge described in the so called “upper-leg”^1^

Indeed, SARS-CoV-1 and SARS-CoV-2 spike structures previously solved by cryo-EM showed one or two RBDs in upward conformation or all the RBDs in the downward conformation (refs. ^2,3^ and references therein; Fig. 1).

Finally, Turoňová et al. succeeded in observing that the stalk emerging from the neck of the spike head connected to the viral membrane appears to be highly dynamic^1^ and contains an 11-residue repeat sequence, enriched in Leucine residues (L1141, L1145, and L1152), adopting an unusual right-handed coiled coil, consistent with a recent single-particle structure of the spike head^1^ defined by the authors as the spike “upper leg”. Right-handed trimeric coiled coils were already seen in the post-fusion structure of the spike protein from the related mouse hepatitis virus (6b3o.pdb),^5^ SARS-CoV-2 spike 3D comparative models,^2^ and the recently SARS-CoV-2 spike monomer fragments built by combining cryo-EM and in silico 3D modeling analysis (6m3w.pdb).

Notably, in a recent analysis of conformational changes determining the post-fusion conformation observed for a similar coronavirus spike cryo-EM structure^5^ and the related SARS-CoV-2 spike 3D comparative model^2^ (Fig. 1), it was proposed that the loss of the N-terminal domain (residues 1–703, black cartoon) causes the reorientation of the 704–715 peptide, which was involved in maintaining the tight packing of the central helix (CH) and the heptad repeat 1 (HR1) domain, together with the N-terminal domain. After the N-terminal cleavage, while HR1 and CH domains relax in the space, the connector domain (CD), consisting of residues 1127–1146, can re-orient itself in the space previously occupied by the removed N-terminal domain, determining the completion of the narrower and elongated spike post-fusion conformation^2^ (Fig. 1).

Thanks to the applied in situ and in silico combined experimental approach, Turoňová et al. analyses allowed to gain new insights about the C-terminal portion orientation and mobility in the prefusion conformation.^1^ Indeed, the atomic coordinates of the C-terminal domain (residues 1147–1288) are not reported in the PDB files of the available solved cryo-EM prefusion structures (i.e., 6vsb.pdb; 6vxx.pdb; 6x2b.pdb; 6vyb.pdb), most likely due to the high flexibility of that protein region. Furthermore, Turoňová et al. got new clues about the mechanism through which the spike C-terminal domain, with the observed three hinges. Confers to the spike head, hosting the RBDs, unexpected orientational freedom, allowing the spike protein to scan the host cell surface for triggering interactions with the human ACE2 receptor.^1–4^

More in detail, visual inspection of the produced subtomograms, in which the stalk domains are clearly observed, corroborated the idea of a kinked stalk with potentially several hinges.^1^ Local refinement of the lower part of the stalk (subsequently referred to as the “lower leg”) and molecular dynamics (MD) simulations confirmed that the spike heads remained stable. The stalks, however, exhibited pronounced hinging motions at the junctions between the spike head and the upper leg (“hip”), between upper and lower leg (“knee”), and between the lower leg and transmembrane domain (“ankle”). Structures of the spike seen along the MD trajectory fit well into the tomographic density of the spike proteins protruding from the viral surface.^1^

Notably, the performed combined analysis allowed also to clarify that RBDs are shielded from antibodies by a glycan coat more extended than expected,^1^ especially when RBDs are in the downward conformation.

In support of what observed by ref.,^1^ it is known that the most performing neutralizing antibodies active against the homologous SARS-CoV spike proteins were able to bind efficiently the spike protein when the RBD subunits in the pre-fusion conformation were in the upward conformation (^2,3^ and references therein).

These observations imply that the flexibility and the orientational freedom conferred by the highlighted hinge regions to SARS-CoV-2 spike prefusion conformation is crucial for a successful infection.

At the same time the glycan coat and the ability to maintain RBDs in downward conformation, most likely depending on the hinging motion of the prefusion conformation C-terminal portion, might represent a further protective strategy for the virus towards neutralizing antibodies.

These observations let us wonder about the need to study new *ad hoc* drugs (i.e., small molecules or peptide drugs, like EK1 previously described for blocking SARS-CoV-1 (5zvm.pdb) and/or SARS-CoV-2 spike HR1 domain (see ref. ^2^ and references therein)) for targeting the described accessible hinge regions^1^ and preventing or limiting conformational changes at the level of the described hinge points, which allow the spike proteins to scan host cell surface, driving conformational changes at the RBD level, for triggering interactions with the human ACE2 receptor.
